# Fat-suppressed gadolinium-enhanced isotropic high-resolution 3D-GRE-T1WI for predicting small node metastases in patients with rectal cancer

**DOI:** 10.1186/s40644-018-0153-9

**Published:** 2018-05-21

**Authors:** Yan Chen, Xinyue Yang, Ziqiang Wen, Baolan Lu, Xiaojuan Xiao, Bingqi Shen, Shenping Yu

**Affiliations:** 1 Department of Radiology, Sun Yat-sen University First Affiliated Hospital, Guangzhou, 510080 China; 2 Department of Radiology, Peking University Shenzhen Hospital, Shenzhen, 518036 China

**Keywords:** Gadolinium-enhanced, 3D-GRE-T1WI, Rectal cancer, Small lymph nodes

## Abstract

**Background:**

To investigate the application value of fat-suppressed gadolinium-enhanced isotropic high-resolution 3D-GRE-T1WI in regional nodes with different short-axis diameter ranges in rectal cancer, especially in nodes ≤5 mm.

**Methods:**

Patients with rectal adenocarcinoma confirmed by postoperative histopathology were included, and all the patients underwent preoperative 3.0 T rectal magnetic resonance imaging (MRI) and total mesorectal excision (TME) within 2 weeks after an MR scan. The harvested nodes from specimens were matched with nodes in the field of view (FOV) of images for a node-by-node evaluation. The maximum short-axis diameters of all the visible nodes in the FOV of images were measured by a radiologist; the morphological and enhancement characteristics of these nodes were also independently evaluated by two radiologists. The *χ*^*2*^ test was used to evaluate differences in morphological and enhancement characteristics between benign and malignant nodes. The enhancement characteristics were further compared between benign and malignant nodes with different short-axis diameter ranges using the *χ*^*2*^ test. Kappa statistics were used to describe interobserver agreement.

**Results:**

A total of 441 nodes from 70 enrolled patients were included in the evaluation, of which 111 nodes were metastatic. Approximately 85.5 and 95.6% of benign nodes were found to have obvious enhancement and homogeneous or mild-heterogeneous enhancement, respectively, whereas approximately 89.2 and 85.1% of malignant nodes showed moderate or mild enhancement and obvious-heterogeneous or rim-like enhancement, respectively. The area under the receiver operating characteristic (ROC) curve (AUC) values of the enhancement degree for identifying the overall nodal status, nodes ≤5 mm and nodes > 5 mm and ≤ 10 mm were 0.887, 0.859 and 0.766 for radiologist 1 and 0.892, 0.823 and 0.774 for radiologist 2, respectively. The AUCs of enhancement homogeneity were 0.940, 0.928 and 0.864 for radiologist 1 and 0.944, 0.938 and 0.842 for radiologist 2, respectively. Nodal border and signal homogeneity were also of certain value in distinguishing metastatic nodes.

**Conclusions:**

Enhancement characteristics based on fat-suppressed gadolinium-enhanced isotropic high-resolution 3D-GRE-T1WI were helpful for diagnosing metastatic nodes in rectal cancer and were a reliable indicator for nodes ≤5 mm.

## Background

Colorectal cancer is the third most common cancer and the fourth leading cause of cancer deaths globally, with rectal cancer accounting for the vast majority of cases [1]. Regional node involvement is associated with local and distant recurrence, along with poor prognosis in rectal cancer [2], and is generally considered an indication for neoadjuvant chemoradiotherapy (CRT) in these patients [3]. Neoadjuvant CRT provides decreased local recurrence and improved general survival, along with less extensive surgery [4]. Therefore, accurate prediction of the regional node status of rectal cancer prior to surgery is closely tied to treatment decisions and prognosis.

The imaging detection of node metastases in rectal cancer is primarily performed with endoluminal ultrasound (EUS), computed tomography (CT) or magnetic resonance imaging (MRI). However, these three modalities have low discriminant accuracy, particularly for nodes smaller than 5 mm [5]. Although high-resolution T2-weighted imaging (T2WI) allows the evaluation of nodal border and signal homogeneity, the diagnostic efficiency has not improved significantly since the majority of metastatic rectal cancer nodes are smaller than 5 mm, making them difficult to evaluate accurately based on morphological changes alone [6]. It is generally believed that gadolinium-enhanced T1-weighted imaging (T1WI) provides minimal benefit for the accurate determination of metastatic nodes in rectal cancer [7]; however, consistent with results of other studies [8–10], gadolinium-enhanced three-dimensional gradient recalled-echo T1-weighted imaging (3D-GRE-T1WI) has shown high accuracy and repeatability in distinguishing malignant from benign nodes in rectal cancer. This technique has been widely used for the head and neck, spine, joints, abdomen, and pelvis [11–15]. However, the discrimination of nodal status in rectal cancer using gadolinium-enhanced 3D-GRE-T1WI has seldom been reported, and a comparative analysis of the diagnostic value of different short-axis diameter ranges has not yet been performed. This study evaluated the enhancement characteristics in lymph nodes and aimed to assess the value of 3.0 T MR fat-suppressed gadolinium-enhanced isotropic high-resolution 3D-GRE-T1WI in the diagnosis of regional node metastases in different short-axis diameter ranges, especially for small nodes in rectal cancer.

## Methods

### Study population

This prospective study was conducted from January 2016 to December 2016. Inclusion criteria consisted of (1) postoperative histopathology confirming primary rectal adenocarcinoma and (2) the existence of 3.0 T rectal MR scans performed with identical imaging parameters within 2 weeks of their curative resection. Exclusion criteria included (1) postoperative histopathology confirming a special histopathological type, such as mucous adenocarcinoma or signet ring cell carcinoma; and (2) history of prior radiotherapy, chemotherapy or other rectal tumor therapies.

### Patient preparation and MR image acquisition

According to the tumor location on colonoscopy, an appropriate amount (20-80 mL) of ultrasonic gel was poured into the rectum but was not used for low or large rectal tumors. To prevent intestinal peristaltic artifacts, a dose of 20 mg of raceanisodamine hydrochloride was injected intramuscularly approximately 10 min prior to the MR examination unless contraindicated.

Imaging was performed using a 3.0 T unit (Magnetom Verio, Siemens, Germany) with a 6-channel phased-array wrap-around surface coil. The coil center was placed on the level of the pubic symphysis and adjusted according to the tumor location. All the patients were placed in the supine position with the feet first. Rectal MRI protocols included high-resolution two-dimensional turbo spin-echo T2-weighted imaging (2D-TSE-T2WI) sequences in the sagittal, coronal and oblique axial planes that were orthogonal to the base of the tumor. In addition, a fat-suppressed gadolinium-enhanced isotropic high-resolution 3D-GRE-T1WI sequence in the coronal plane was applied. Details of the protocols are listed in Table 1. The technique for water excitation normal was employed to suppress fat. Gadolinium (Gadopentetate Dimeglumine Injection, Consun, Guangzhou) was administered at a dose of 0.2 mL/kg bodyweight and a rate of 3.0 mL/sec by a bolus injection with a power injector through the cubital vein. Then, a dose of 25 mL of 0.9% saline was injected at the same rate.

**Table 1:** Rectal high-resolution MRI protocols for 2D and 3D sequences

Sequences	TR/TE (msec)	Slice thickness/Gap (mm)	No. of slices	Frequency direction	Flip angle (°)	Matrix	FOV (cm)	Voxel size (mm)	Acquisition time
2D-TSE-T2WI									
Sagittal	3000/87	3/0	19	H to F	150	320 × 256	18	0.7 × 0.6 × 3.0	2 min 30 s
Coronal	4000/77	3/0	25	F to H	137	384 × 308	22	0.7 × 0.6 × 3.0	2 min 52 s
Oblique axial	3000/84	3/0	24	R to L	150	320 × 320	18	0.6 × 0.6 × 3.0	3 min 18 s
3D-GRE-T1WI									
Coronal	10/4.9	1/0	144	R to L	10	384 × 384	38	1.0 × 1.0 × 1.0	3 min 10 s

Notes: *TR* repetition time, *TE* echo time, *FOV* field of view, *H* head, *F* feet, *R* right, *L* left

### Image evaluation

Two radiologists (R1: YC, R2: XY), who were experienced in reading MR images of rectal cancer and were blind to the histopathological results, analyzed all the rectal MR images at the MRI workstation.

First, each visible node was carefully identified in the field of view (FOV) of MR images by one radiologist (R1). Meanwhile, the maximum short-axis diameters (millimeters) of the nodes were measured three times with a workstation electronic caliper, and the average readings were reported. Then, two radiologists independently evaluated and recorded the nodal border and signal homogeneity on high-resolution 2D-TSE-T2WI images, in addition to the enhancement degree and homogeneity on fat-suppressed gadolinium-enhanced isotropic high-resolution 3D-GRE-T1WI images.

**Fig. 1:**
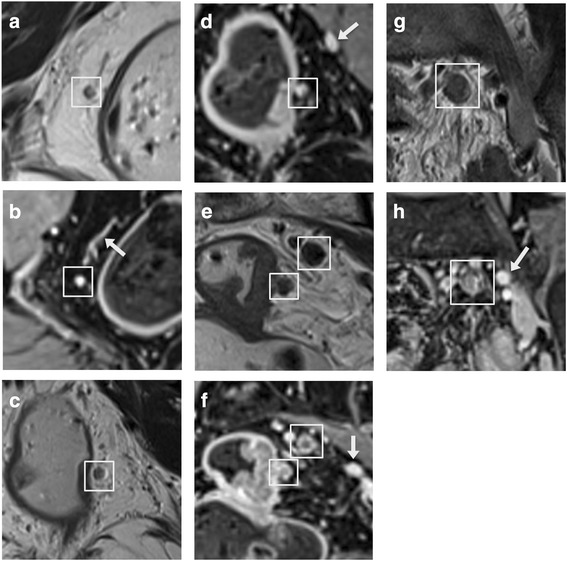
The white boxes indicate lymph nodes, and the white arrows indicate vessels. (**a**, **c**, **e** and **g**), coronal high-resolution 2D-TSE-T2WI; (**b**, **d**, **f** and **h**), coronal fat-suppressed gadolinium-enhanced isotropic high-resolution 3D-GRE-T1WI. **a**-**b**, Benign node 3.0 mm in diameter with a smooth border, homogeneous signal, and obvious and homogeneous enhancement. **c-d**, Benign node 3.6 mm in diameter with a smooth border, mild-heterogeneous signal, and obvious and mild-heterogeneous enhancement. **e-f**, Node 4.2 mm in diameter adjacent to the rectal wall that was malignant with an irregular border, mild-heterogeneous signal, and intermediate and rim-like enhancement. The superior node 6.2 mm in diameter was also malignant, with a smooth border, obvious-heterogeneous signal, and mild and rim-like enhancement. **g**-**h**, Malignant node 8.2 mm in diameter with an irregular border, mild-heterogeneous signal, and mild and obvious-heterogeneous enhancement

These evaluation parameters were defined specifically as follows: (1) the nodal border was categorized as either smooth or irregular; (2) the signal homogeneity was classified as homogeneous, mild-heterogeneous or obvious-heterogeneous; (3) the enhancement degree was categorized into three subtypes by comparison to the vessels at the same level, and obvious enhancement was recorded if the node appeared to be equal in signal intensity, mild enhancement was recorded if the node appeared to have significantly low signal intensity, and intermediate enhancement was recorded if the signal intensity was between obvious and mild enhancement. When a node appeared to have heterogeneous enhancement, the enhancement degree of the main region in the node was evaluated; (4) the enhancement homogeneity was classified as homogeneous, mild-heterogeneous, obvious-heterogeneous or rim-like enhancement [10, 16] (Fig. 1).

### Histopathologic assessment and nodal comparison

All the enrolled patients underwent total mesorectal excision (TME) of the rectum within 2 weeks after MR scans (median duration: 5 days; range: 1-14 days). On the basis of localized nodes that were all visible in the FOV on preoperative MR images, a surgeon with expertise in colorectal cancer successively localized and recorded regional nodes in different groups during surgery. Then, these nodes were removed from the specimen and taken to the pathology department promptly placed in individual trays marked according to each node identified by MR images. All the nodes were analyzed by a dedicated gastrointestinal pathologist and reported as malignant when tumor cells were observed in the node under a light microscope. To provide an accurate node-by-node comparison of MR images and histopathologic findings, special attention was paid to the nodal size and morphology, in addition to the position of the node relative to the tumor, rectal wall, mesorectal fascia, vessels and adjacent nodes. The nodes were matched with MR images in corresponding groups and were excluded if they could not be matched. The pathological staging of rectal cancer referred to the rules for TNM staging of the American Joint Committee on Cancer (AJCC)/ Union for International Cancer Control (UICC) [17].

### Statistical methods

Statistical analysis was performed using SPSS 20.0 software. All quantitative data were tested for a normal distribution with the one-sample Kolmogorov-Smirnov test. Quantitative data with a non-Gaussian distribution were expressed as the medians with the ranges. Correlation analysis was performed with the Spearman rank correlation test. The *χ*^*2*^ test was used to compare the correlated qualitative factors (nodal border, signal homogeneity, enhancement degree and homogeneity) between benign and malignant nodes. Kappa statistics (0.00-0.20 poor, 0.21-0.40 fair, 0.41-0.60 moderate, 0.61-0.80 good and 0.81-1.00 excellent agreement) were calculated for the evaluation of interobserver agreement. Clinical pathological features were also compared between node-negative and node-positive patients using the *χ*^*2*^ test or the Mann-Whitney *U* test. A two-tailed *P* value < 0.05 was considered to indicate a statistically significant difference. Receiver operating characteristic (ROC) analyses were performed to assess the diagnostic utility of the enhancement characteristics for the detection of metastatic nodes in different short-axis diameter ranges, and the area under the ROC curve (AUC) and 95% confidence interval (CI) were calculated. Each AUC value was interpreted as having no (< 0.5), low (0.5-0.7), moderate (0.7-0.9) or high (> 0.9) diagnostic value. As mentioned above, the morphological characteristics of small nodes are currently difficult to observe well on high-resolution MRI. Therefore, the morphological characteristics of nodes in different short-axis diameter ranges (≤5 mm, > 5 mm and ≤ 10 mm, and > 10 mm) were not further analyzed in this study.

## Results

### General and histopathological findings

A total of 70 patients (36 males and 34 females; median age: 60 years; range: 31-80 years) were enrolled in this study. Of the 70 patients, 36 (51.4%) were confirmed to have metastatic regional nodes. Histopathology of 1004 nodes harvested from the rectal specimens in 70 patients (median: 13; range: 7-45) indicated that 176 (17.5%) contained metastases. Lymph node metastases were more likely occur in pT3-4 patients (*P* < 0.001). The location and differentiation of rectal cancer were not related to whether the patient had metastatic nodes (*P* = 0.055, 0.052, respectively) (Table 2).

**Table 2:** Relationship between clinical pathological features and nodal metastases in 70 patients

Parameters	Patients with nodal metastases			*P*
	Total (*n* = 70)	Negative (*n* = 34)	Positive (*n* = 36)	
Age,median (range)	60 (31-80)	60 (31-76)	61 (42-80)	0.155
Gender				0.469
Male	36 (51.4%)	19 (55.9%)	17 (47.2%)	
Female	34 (48.6%)	15 (44.1%)	19 (52.8%)	
Location				0.055
Low	33 (47.1%)	21 (61.8%)	12 (33.3%)	
Middle	27 (38.6%)	10 (29.4%)	17 (47.2%)	
High	10 (14.3%)	3 (8.8%)	7 (19.4%)	
Differentiation				0.052
Well	2 (2.9%)	2 (5.9%)	0 (0.0%)	
Moderately	56 (80.0%)	29 (85.3%)	27 (75.0%)	
Poorly	12 (17.1%)	3 (8.8%)	9 (25.0%)	
T stage				< 0.001
pT1	5 (7.1%)	5 (14.7%)	0 (0.0%)	
pT2	16 (22.9%)	13 (38.2%)	3 (8.3%)	
pT3	22 (31.4%)	9 (26.5%)	13 (36.1%)	
pT4	27 (38.6%)	7 (20.6%)	20 (55.6%)	

Notes: According to the distance from the most caudal border of the rectal tumor to the anal verge on MRI: low, < 5 cm; middle, 5-10 cm; high, > 10 cm; *p* pathological

For the node-by-node evaluation, a correlation between the results of MR images and histopathology was feasible for 441 (43.9%) nodes, including 111 metastatic nodes. According to the MR measurements, the median short-axis diameter 3.8 mm (range: 1.2-18.1 mm) for all the nodes; 3.4 mm (range: 1.2-8.4 mm) for the benign nodes; and 7.0 mm (range: 3.1-18.1 mm) for the malignant nodes. Of 313 nodes with short-axis diameter ≤ 5 mm, 23 (7.3%) contained metastases; of 111 nodes > 5 mm and ≤ 10 mm, 71 (64.0%) contained metastases; and all 17 nodes > 10 mm contained metastases.

### Relationship between nodal status and its morphological and enhancement characteristics (Table 3)

**Fig. 2:**
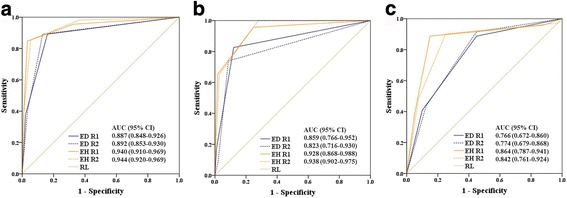
ROC curves and AUCs of enhancement characteristics for determining nodal status for nodes (**a**), overall; (**b**), ≤5 mm; and (**c**), > 5 mm and ≤ 10 mm. Notes: *ED*  enhancement degree; *EH*  enhancement homogeneity; *RL*  reference line; *R*  radiologist

Malignant nodes mostly demonstrated an irregular border (R1: 73.9%, R2: 70.3%) and a mild-heterogeneous or obvious-heterogeneous signal (R1: 92.8%, R2: 90.1%). Additionally, 83.9 and 87.0% of benign nodes showed obvious enhancement according to the two radiologists; conversely, only 10.8% of metastases were found to have obvious enhancement, and most (89.2% for both radiologists) had mild or intermediate enhancement. Regarding enhancement homogeneity, benign nodes were more commonly (R1: 96.7%, R2: 94.5%) found to have homogeneous or mild-heterogeneous enhancement, whereas 84.7 and 85.5% of malignant nodes were detected to have obvious-heterogeneous or rim-like enhancement by the two radiologists. The larger nodes were, the more heterogeneous the signal (R1: *r*_*s*_ = 0.639, *P* < 0.001, R2: *r*_*s*_ = 0.720, *P* < 0.001) and enhancement were (R1: *r*_*s*_ = 0.757, *P* < 0.001, R2: *r*_*s*_ = 0.785, *P* < 0.001). The interobserver agreement values for nodal border, signal homogeneity, enhancement degree and homogeneity were good (*κ* = 0.633, 0.611, 0.703 and 0.744, respectively). The ROC curve of enhancement characteristics for the prediction of nodal status is shown in Fig. 2a.

**Table 3:** Rectal cancer nodal morphological and enhancement characteristics on MR images versus histopathological findings in 440 nodes

Radiologist	Radiologist 1			Radiologist 2			*κ*
Histopathologic Findings	Benign (330)	Malignant (111)	*P*	Benign (330)	Malignant (111)	*P*	
Border			< 0.001			< 0.001	0.633
Smooth	319 (96.7%)	29 (26.1%)		305 (92.4%)	33 (29.7%)		
Irregular	11 (3.3%)	82 (73.9%)		25 (7.6%)	78 (70.3%)		
Signal homogeneity			< 0.001			< 0.001	0.611
Homogenous	230 (69.7%)	8 (7.2%)		225 (68.2%)	11 (9.9%)		
Mild-heterogeneous	99 (30.0%)	46 (41.4%)		104 (31.5%)	59 (53.2%)		
Obvious-heterogeneous	1 (0.3%)	57 (51.4%)		1 (0.3%)	41 (36.9%)		
Enhancement degree			< 0.001			< 0.001	0.703
Obvious	277 (83.9%)	12 (10.8%)		287 (87.0%)	12 (10.8%)		
Intermediate	46 (13.9%)	57 (51.4%)		32 (9.7%)	55 (49.5%)		
Mild	7 (2.1%)	42 (37.8%)		11 (3.3%)	44 (39.6%)		
Enhancement homogeneity			< 0.001			< 0.001	0.747
Homogeneous	223 (67.6%)	5 (4.5%)		210 (63.6%)	2 (1.8%)		
Mild-heterogeneous	96 (29.1%)	12 (10.8%)		102 (30.9%)	14 (12.6%)		
Obvious-heterogenous	2 (0.6%)	36 (32.4%)		3 (0.9%)	42 (37.8%)		
Rim-like	9 (2.7%)	58 (52.3%)		15 (4.5%)	53 (47.7%)

### Relationship between nodal status and its enhancement characteristics in subgroups (Table 4)

Among nodes with short-axis diameter ≤ 5 mm, 87.9 and 91.4% of benign nodes showed obvious enhancement, compared with 82.6 and 73.9% of malignant nodes showed mild or intermediate enhancement. Additionally, 98.3 and 97.3% of benign nodes showed homogeneous or mild-heterogeneous enhancement, and 65.2% of malignant nodes were found to have obvious-heterogeneous or rim-like enhancement. The interobserver agreement value for enhancement homogeneity was good (*κ* = 0.699), whereas that for enhancement degree was moderate (*κ* = 0.593).

**Table 4:** Rectal cancer nodal enhancement characteristics on MR images versus histopathological findings in subgroups with different short-axis diameter ranges

Radiologist	Radiologist 1			Radiologist 2			*κ*
Histopathologic Findings	Benign	Malignant	*P*	Benign	Malignant	*P*	
≤5 mm	290	23		290	23		
Enhancement degree			< 0.001			< 0.001	0.593
Obvious	255 (87.9%)	4 (17.4%)		265 (91.4%)	6 (26.1%)		
Intermediate	32 (11.0%)	15 (65.2%)		19 (6.6%)	15 (65.2%)		
Mild	3 (1.0%)	4 (17.4%)		6 (2.1%)	2 (8.7%)		
Enhancement homogeneity			< 0.001			< 0.001	0.699
Homogeneous	218 (75.2%)	1 (4.3%)		209 (72.1%)	0 (0.0%)		
Mild-heterogeneous	67 (23.1%)	7 (30.4%)		73 (25.2%)	8 (34.8%)		
Obvious-heterogenous	0 (0.0%)	3 (13.0%)		0 (0.0%)	1 (4.3%)		
Rim-like	5 (1.7%)	12 (52.2%)		8 (2.8%)	14 (60.9%)		
> 5 mm and ≤ 10 mm	40	71		40	71		
Enhancement degree			< 0.001			< 0.001	0.627
Obvious	22 (55.0%)	8 (11.3%)		22 (55.0%)	6 (8.5%)		
Intermediate	14 (35.0%)	34 (47.9%)		13 (32.5%)	34 (47.9%)		
Mild	4(10.0%)	29 (40.8%)		5 (12.5%)	31 (43.7%)		
Enhancement homogeneity			< 0.001			< 0.001	0.651
Homogeneous	5 (12.5%)	3 (4.2%)		1 (2.5%)	2 (2.8%)		
Mild-heterogeneous	29 (72.5%)	5 (7.0%)		29 (72.5%)	5 (7.0%)		
Obvious-heterogenous	2 (5.0%)	27 (38.0%)		3 (7.5%)	34 (47.9%)		
Rim-like	4 (10.0%)	36 (50.7%)		7 (17.5%)	30 (42.3%)		
> 10 mm*	0	17		0	17		
Enhancement degree			–			–	0.521
Obvious	0	0 (0.0%)		0	0		
Intermediate	0	8 (47.1%)		0	6 (35.3%)		
Mild	0	9 (52.9%)		0	11 (64.7%)		
Enhancement homogeneity			–			–	0.783
Homogeneous	0	1 (5.9%)		0	0		
Mild-heterogeneous	0	0 (0.0%)		0	1 (5.9%)		
Obvious-heterogenous	0	6 (35.3%)		0	7 (41.2%)		
Rim-like	0	10 (58.8%)		0	9 (52.9%)		

Notes: *The *χ*^*2*^ test was not used in the subgroup with > 10 mm nodes because all were metastatic; that is, the dependent variable was constant

Among nodes with short-axis diameter > 5 mm and ≤ 10 mm,55.0% of benign nodes showed obvious enhancement, and 88.7 and 91.6% of metastatic nodes showed mild or intermediate enhancement. Additionally, 85.0 and 75.0% of benign nodes manifested homogeneous or mild-heterogeneous enhancement, while 88.7 and 90.2% of malignant nodes showed obvious-heterogeneous or rim-like enhancement. The interobserver agreement values for enhancement degree and homogeneity were good (*κ* = 0.627 and 0.651, respectively).

The nodes > 10 mm were all metastatic and all showed mild or intermediate enhancement, and 94.1% showed obvious-heterogeneous or rim-like enhancement. The interobserver agreement value for enhancement homogeneity was good (*κ* = 0.783), whereas that for enhancement degree was moderate (*κ* = 0.521).

Subgroup ROC curves of the enhancement characteristics for the prediction of nodal status are shown in Fig. 2b, c.

## Discussion

High-resolution pelvic MRI is widely considered the optimal imaging method for rectal cancer [3], and it enables precise identification of high-risk factors, such as the extent of tumor invasion, extramural vascular invasion (EMVI) and the potential circumferential resection margin (CRM) [18]; however, the prediction of regional node metastases remains a challenge. Size is the usual criterion for metastatic nodes using MRI; however, the considerable size overlap between benign and malignant nodes affects the overall predictive value [19]. We found similar results in this study. The short-axis diameters of benign and malignant nodes ranged from 1.2 to 8.4 mm and from 3.1 to 18.1 mm, respectively. Discrimination between benign and malignant nodes by high-resolution MRI may be more reliable than that by nodal size when morphologic features such as border and signal homogeneity are also considered [16, 20]. However, due to the limitation of image acquisition resolution and differences in image feature interpretation, the consistency between observers may be poor [21]; moreover, the ability to resolve such small nodes is apparently suboptimal [22]. Most studies [7, 23] have suggested that MRI with an intravenous gadolinium-based contrast agent did not improve the accuracy of metastatic nodal diagnosis in rectal cancer. However, Beets-Tan’s [8–10] team reported that assessing nodes using gadolinium-enhanced 3D-GRE-T1WI with a 1.5 T unit not only found a better nodal detection rate and better observation of nodal characteristics but also produced a powerful predictor of nodal status that showed satisfactory reproducibility.

Fat-suppressed gadolinium-enhanced isotropic high-resolution 3D-GRE-T1WI serves as a promising technique for rectal metastatic node detection. This technique adopts multiple approaches of fast acquisition to cover the entire pelvis, contributing to a decrease in the risk of motion artifacts. In addition, this technique maintains a higher signal-to-noise ratio (SNR) despite its thinner slice thickness due to the lack of interlayer interference and phase-encoding direction oversampling in all three sections. Moreover, this technique provides much higher spatial resolution than a 2D high-resolution sequence and can provide multiplanar reconstruction (MPR) images at an arbitrary angle from a no-interval volumetric interpolated examination of thinner thicknesses. Additionally, the sequential symmetric k-space filling technique can ensure its centric contrast and retain the surrounding datum, which not only provides better-detailed anatomical structures but also increases the vessel contrast. Furthermore, lymph nodes are generally distributed along vessels and embedded in fat tissue around the vessels. The above-mentioned advantages not only can provide easier detection of small nodes and differentiation from surrounding vessels but can also yield higher contrast than either high-resolution T2WI or the common enhanced sequence; therefore, nodal enhancement characteristics can be easily depicted [12, 24, 25]. This study found that fat-suppressed gadolinium-enhanced isotropic high-resolution 3D-GRE-T1WI could ensure more accurate identification of rectal metastatic from benign nodes ≤5 mm than an assessment using high-resolution T2WI based on morphology (AUC: 0.72-0.77) or the common enhanced scan (AUC: 0.70-0.80) [7]. Enhancement degree and homogeneity were satisfactory criteria for evaluating the status of such small nodes, with moderate to high AUCs (R1: 0.859 and 0.928, respectively, R2: 0.823 and 0.938, respectively), and the results were comparable to those of Zhang et al. [26], who assessed nodes ≤5 mm using high-resolution T2WI on the strength of the chemical shift effect (AUC: 0.845-0.879).

Enhancement characteristics were proven to be highly reliable predictors of nodal positivity, with a significant difference between benign and malignant nodes in different short-axis diameter ranges. These characteristics tended to show obvious enhancement in benign nodes (R1: 83.9%, R2: 87.0%) and mild or intermediate enhancement in malignant nodes (89.2% for both radiologists). Nearly all (R1: 96.7%, R2: 94.5%) the benign nodes manifested homogeneous or mild-heterogeneous enhancement; in contrast, quite a few (R1: 84.7%, R2: 85.5%) metastatic nodes showed obvious-heterogeneous or rim-like enhancement. The larger nodes became prone to necrosis and liquefaction; hence, they had a tendency toward a more heterogeneous signal. Thus, obvious-heterogeneous or rim-like enhancement was usually (94.1% for both radiologists) observed in nodes > 10 mm. These results essentially met the pathophysiological basis of contrast material uptake by lymph nodes. Normal and reactive nodes would take up gadolinium-based medium, resulting in an intense enhancement comparable to that of the surrounding vessels. Conversely, in metastatic nodes, normal lymphatic tissue and macrophages within sinuses were replaced by the tumor to varying degrees, hindering the medium uptake. Therefore, malignant nodes had a significantly longer time to peak and showed mild and heterogeneous enhancement [27–29].

However, it should be noted that some of the nodes had atypical enhancement characteristics. (1) For example, 10.8% of metastatic nodes showed obvious enhancement. This finding likely occurred because intravenously injected contrast medium entered medullary sinuses directly via capillary interendothelial channels. However, if the contrast medium nonspecifically permeated into the interstitial space through fenestrated capillaries and was then transported to nodes via lymphatic vessels, the medium would be prevented from entering nodes by tumor cells in the interstitial spaces or nodes [28]. (2) More than half of the nodes located in the presacral space along the superior rectal vessels contained dilated vessels, thus possibly causing rapid gadolinium washout [8]. This situation could be responsible for the benign nodes in this area showing low enhancement. (3) The homogeneous or mild-heterogeneous enhancement in 15.3 and 14.4% of malignant nodes may be related to an inability to discern nodal micrometastases, although our study employed the isotropic 3D technique with 1 mm slice thickness without interslice spacing. (4) Some benign nodes were detected with mild-heterogeneous enhancement, probably because of the limitation of partial volume averaging effects or the non-uniform distribution of capillary density within nodes [30].

Our results showed that 26.1 and 29.7% of metastatic nodes exhibited a smooth border. However, most of these malignant nodes showed mild or intermediate and/or obvious-heterogeneous or rim-like heterogeneous features. This finding may have occurred because the tumor within a node that had not yet infiltrated the peripheral capsule into extranodal fat would have smooth nodal borders, whereas tumor cells inside the nodes influenced the uptake of gadolinium, leading to their enhancement characteristics differing from those of benign nodes. It was suggested that enhancement characteristics seem to be more sensitive than border status for identifying nodal status. A relatively large number of nodes with a mild-heterogeneous signal were found for each of the two nodal statuses (R1: 30.0% vs. 41.1%, R2: 31.5% vs. 53.2%). This finding might be correlated with the use of high-resolution MRI, which shows different signal intensities of the anatomic structures inside a benign node rather than showing uniform signal intensity on non-high-resolution images. Most of the nodes were ≤ 5 mm, which may have led to the misinterpretation of internal signal characteristics due to the limited spatial resolution.

The result showing increased risk of nodal metastases with higher tumor stages was in line with previous studies [21]. This finding may be due to lymphatic vessels being mainly located in the submucosa; thus, deeper infiltration of the tumor will correspond to a greater likelihood that the nodes will be affected [31].

There are some potential limitations of this study. First, the nodal match rate was not very high. This rate was mainly affected by the presence of a degree of specimen distortion and the harvesting of some nodes from specimens out of the FOV of MR images. Second, the number of metastatic nodes was relative lower than that of benign nodes, and for patients with definite metastatic nodes, neoadjuvant CRT is usually recommended. Third, the numbers of nodes in different short-axis diameter ranges was a far cry, especially such little nodes > 10 mm. Statistical analyses could not be conducted for all the evaluated malignant nodes. Fourth, iliac nodes were not assessed because TME is not typically performed for an extended pelvic lymphadenectomy. Finally, a multicenter study should be conducted to further evaluate the clinical significance of the gadolinium-enhanced isotropic high-resolution 3D sequence. Confirming the value of this sequence in a large patient cohort would make a crucial impact on the patient selection for personalized treatment. Patients with node-positive disease will benefit from neoadjuvant CRT, whereas true node-negative patients may undergo immediate surgery [3, 4].

## Conclusions

Enhancement characteristics based on fat-suppressed gadolinium-enhanced isotropic high-resolution 3D-GRE-T1WI yielded better predictive power for regional nodes in rectal cancer and hold considerable promise for determining the status of nodes ≤5 mm. Nodal border and signal homogeneity also provided some contribution but were not as powerful as enhancement characteristics. These findings suggest prospects for the broad application of these enhanced observations to 3D sequence.
